# Investigating the Dynamics of China’s Green Building Policy Development from 1986 to 2019

**DOI:** 10.3390/ijerph18010196

**Published:** 2020-12-29

**Authors:** Zezhou Wu, Qiufeng He, Kaijie Yang, Jinming Zhang, Kexi Xu

**Affiliations:** 1 Sino-Australia Joint Research Center in BIM and Smart Construction, Shenzhen University, Shenzhen 518060, China; wuzezhou@szu.edu.cn (Z.W.); 1900474011@email.szu.edu.cn (Q.H.); 2060474011@email.szu.edu.cn (K.Y.); 2 School of Political Studies, Nanjing Agricultural University, Nanjing 210095, China; 3 School of Public Administration, Zhejiang University of Finance and Economics, Hangzhou 310018, China; xkxzj2017@zufe.edu.cn

**Keywords:** green building, policy evolution, bibliometric analysis, text mining, China

## Abstract

China has enacted numerous green building policies (GBPs) to promote green building (GB) development in the past decades. Investigating the evolution characteristics of China’s GBPs is significant for the future optimization of the GBP system. However, few studies on this topic have been conducted. To bridge this research gap, this paper adopted the methods of bibliometric analysis and text mining to probe the dynamic evolution of the GBPs in China. Firstly, a total 199 collected policies from 1986 to 2019 were grouped into five stages according to the Five-Year Plan. Then, the topics emphasized in different stages and the cooperative relationships among policymaking agencies were discovered by mapping and visualizing the co-word network and co-author network. Based on the derived results, an in-depth discussion was further conducted from five aspects: targets, objects, instruments, GB performance indicators, and the collaboration structure of policymaking agencies. It was revealed that the topics of GBPs evolved from macro to specific, and the types of policy targets, objects, instruments, and GB performance indicators evolved from few to multiple. Additionally, the collaboration structure of policymaking agencies went from dispersive to centralized. This study sheds lights on the dynamic evolution of China’s GBPs and provides valuable references for other countries in need.

## 1. Introduction

Due to the overexploitation and uncontrolled use of resources, global resources are being consumed at an alarming rate and the global environment is being seriously damaged [1,2,3]. At present, the world is faced with increasing greenhouse gas emissions, reducing forest cover, diminishing biodiversity, and depleting freshwater systems and natural resources [4,5,6,7,8,9,10]. To some extent, the building sector should be responsible for such resource abuse and environmental damage because it has brought a lot of negative impacts to society, including waste of building materials, dust production, water pollution, high energy consumption, harmful gases, and so on [11,12,13,14,15,16]. Against this backdrop, there are more and more calls to promote the sustainable development of the building sector, and green building (GB) came into being due to its advantages of minimizing the negative impacts on the environment and improving the living conditions of occupants [17,18,19]. Given these advantages, GB has been advocated and promoted all over the world as a guiding paradigm for the sustainable development of the building sector [20,21].

Green building policies (GBPs) are regarded as playing an important role in promoting GB practice [22,23]. In China, the government has issued numerous policies to promote green building development. As early as 1986, the promulgation of the “Design Standard for Energy-Saving of Civil Buildings (Heating Residential Buildings)” marked the beginning of GBP formulation in China. Since then, to better guide and support green building practice, the Chinese government has been constantly improving the GBP system and shifting the topics of GBPs [24]. For instance, the focus of the topic “GB technology” shifted several times over the past decades, from the new wall material technology in 1992 to resource utilization of construction waste and prefabricated technology in 2013 [25]. In addition, in terms of the green building assessment standard, the focus turned from emphasizing resource saving and environmental protection technologies to people-oriented aspects [26].

As the topics of GBPs keep shifting, a clear understanding of the policy evolution dynamics is essential for stakeholders to better grasp the key points of green building development. For GB practitioners, understanding the dynamics of GBPs can help them to address the most innovative GB technologies and the most recent incentive measures. For policymakers, the analysis of the collaboration structures can facilitate better understanding the distribution of responsibility in different government departments with respect to policy design. In addition, since China owns one of the largest construction industries in the world [27], a study of the dynamic evolution of China’s GBPs can offer a valuable reference for other countries in need.

In existing literature, however, few studies have attempted to systematically investigate the dynamics of GBPs in China. Some existing studies just investigated green building policy in a short historical period (e.g., the 11th Five-Year Plan period) or mainly focused on specific policies (e.g., green retrofit policies). For instance, Shi et al. [28] evaluated the effectiveness of green building policies during the 11th Five-Year Plan period. Liu et al. [29] reviewed China’s green retrofit policies during 1996–2019 and identified the deficiencies of the current policy system. Ye et al. [30] investigated more than 70 green building standards in China and proposed development suggestions for green building standards. To bridge this research gap, this paper conducts a bibliometric analysis and text mining to investigate the dynamics of China’s national-level GBPs from 1986 to 2019.

The data collection and analysis methods are described in Section 2. Then, the topics in different stages and the collaboration structures of government departments are analyzed in Section 3. Following the analysis results, China’s GBPs are discussed from five aspects (e.g., policy targets, objects, instruments, GB performance indicators, and the collaboration structure of policymaking agencies) in Section 4. Finally, a conclusion is given in Section 5.

## 2. Materials and Methods

### 2.1. Data Collection

The policy documents investigated in this research were retrieved from three official websites and two well-known databases. The three official websites included the Central Government, the Ministry of Housing and Urban-Rural Development (MOHURD), and the National Development and Reform Commission, which are usually used to publish national-level policies related to the building sector [31]. The two databases, namely, the Magic Weapon of Peking University and the China’s National Knowledge Infrastructure database [32], were selected to serve as supplementary retrieval databases to ensure the integrity of data collection. The two databases were chosen because they are the largest policy database and the largest academic database in China, respectively. 

**Figure 1:**
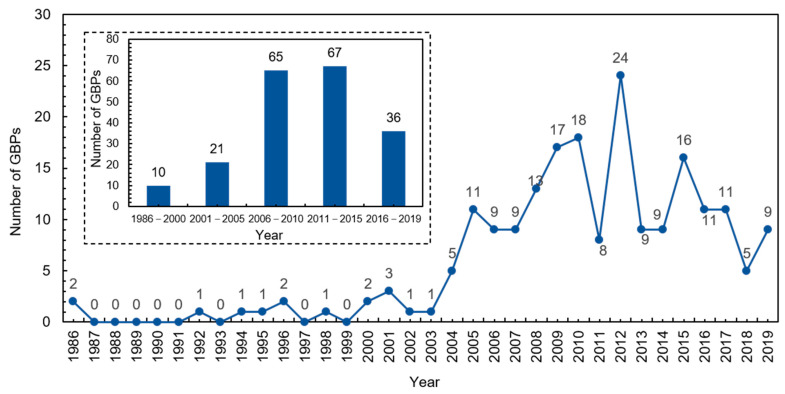
Distribution of China’s green building policies (GBPs) from 1986 to 2019.

According to the release date of China’s first GBP, the retrieval time range was determined as being from 1 January 1986 to 31 September 2019. The search keywords were retrieved from numerous review literature on GB [33,34,35,36,37] covering green buildings, ecological buildings, sustainable buildings, high-performance buildings, green building technology, and green construction. The keywords were applied to the content of policy and the document type was set at the national level. Initially, a total of 557 policy documents were collected from all websites and databases. A GBP document usually constitutes a title and contents. As the search keywords were applied to the policy content, it was possible that some of the retrieved documents just mentioned those keywords rather than explaining or introducing them in detail. Thus, a manual check was subsequently conducted to filter the initially collected policies according to the following criteria: (1) The policies that were duplicates or had no substantive information related to GB were eliminated. (2) The policies needed to be in the form of a law, regulation, measure, notice, opinion, or other document representing government policy, excluding news reports or government daily work report documents. (3) The policy needed to be a national-level policy issued by the central government or its directly affiliated agencies. Ultimately, 199 national GBPs were obtained for policy analysis. The number distribution of the identified GBPs is shown in Figure 1.

From Figure 1, it can be seen that before 2004, the number of GBPs published each year was less than four. However, since 2004, the number of GBPs has increased steadily, with five or more GBPs published each year. According to the number of policies published each year and the Five-Year Plan, five stages (i.e., 1986–2000, 2001–2005, 2006–2010, 2011–2015, and 2016–2019) were grouped for further analysis. The Five-Year Plan was selected because it is regarded one of the most important national policies in China and has been successfully applied in other policy studies [38,39].

### 2.2. Research Methods

#### 2.2.1. Bibliometric Analysis

Bibliometric analysis employs a quantitative and visual processes approach for the description, evaluation, and monitoring of published research to measure scientific progress and production results in a specific field over a period of time [40]. This method has been applied in some previous studies related to policy analysis, and its effectiveness has been well confirmed [41,42,43]. Compared with qualitative research, bibliometric analysis reduces the dependence on researchers’ knowledge and experience and makes the research results more repeatable and verifiable [31,41]. Bibliometric analysis mainly includes five specific methods: bibliographical coupling, co-citation analysis, citation analysis, co-author analysis, and co-word analysis [40,44]. The latter three methods are the most commonly employed in policy analysis. Citation analysis is usually applied to evaluate the impact of policies [43]. If a policy is heavily cited, it is considered to be important [40]. Co-author analysis uses co-authorship data to reveal the collaborative relationship between policymaking agencies [42]. Co-word analysis is usually utilized in conjunction with social network analysis to capture the historical dynamics of policy topics through the keywords in the policy [43]. In this study, co-word analysis and co-author analysis are adopted to analyze China’s GBPs. 

Co-word analysis is a content analysis technique that uses the keywords in documents to establish relationships and builds a conceptual structure of the domain [45,46]. This method has been used to search management information systems, analyze research trends [47], discover research hotspots [48], and identify the evolution of research topics [49]. Generally, co-word analysis of policy documents includes three steps [50]: (1) extracting 3–6 keywords from policy documents; (2) utilizing Bibexcel to establish a co-word matrix; (3) adopting the social network analysis to establish a modular matrix; and (4) employing Gephi software to generate a visualization network of the keywords by running its Layout module and Modularity module [51,52]. 

Co-author analysis explores the cooperative relationships among policymaking agencies in the release of policy documents, similar to the steps of co-word network analysis. Firstly, Bibexcel was utilized to establish the frequency statistics of policymaking agencies and to generate their co-occurrence matrix. Then, Gephi software was utilized to generate a visualization graph to clarify cooperative relationships among policymaking agencies.

#### 2.2.2. Text Mining

Text mining is considered to be an effective solution to extract keywords from documents [43,53]. A three-sub-step approach was used in this step. The first step was to separate words. Based on the Jieba package in Python, a series of sentences was separated into individual words to reduce the dimension of the computer processing text. Nevertheless, some technical terms were not expected to be separated. It was expected that the term “green building” be presented in this way, instead of “green” and “building.” Thus, the custom dictionary was implemented in this step. The second step was to remove stop words, which are meaningless and are frequently used in the document, such as “the first item” and “increase strength.” Ultimately, term frequency–inverse document frequency (TF–IDF) was utilized to extract keywords for each document, which is an effective method to capture words that do not emerge frequently but are uniquely representative in different documents [54]. The specific calculation process of TF–IDF is shown in Equations (1)–(3) [55]:

(1) $$tf_{ij}=\frac{n_{i,j}}{\sum_{k}n_{k,j}}$$

(2) $$idf_{i}=log\frac{\left|D\right|}{\left|1+\left\{j:t_{i}\in d_{j}\right\}\right|}$$

(3) $$TF-IDF=tf_{i,j}\times idf_{i,j}$$

In Equation (1), *i* is a specific word, *j* is a document containing the word *i*, $n_{i,j}$ represents the number of times the word *i* appears in document *j*, and $\underset{k}{\sum }n_{k,j}$ is the sum of the occurrences of all the words in document *j*.

In Equation (2), |D| is the total number of documents and $\left\{j:t_{i}\in d_{j}\right\}$ represents the number of documents containing the word *i*. 

Equation (3) is the product of $tf_{i,j}$ with $idf_{i,j}$, where $tf_{i,j}$ is the frequency that word *i* appears in document *j*, and $idf_{i,j}$ is the frequency that word *i* appears in all documents.

## 3. Results

### 3.1. GBP Topics in Different Stages

**Figure 2:**
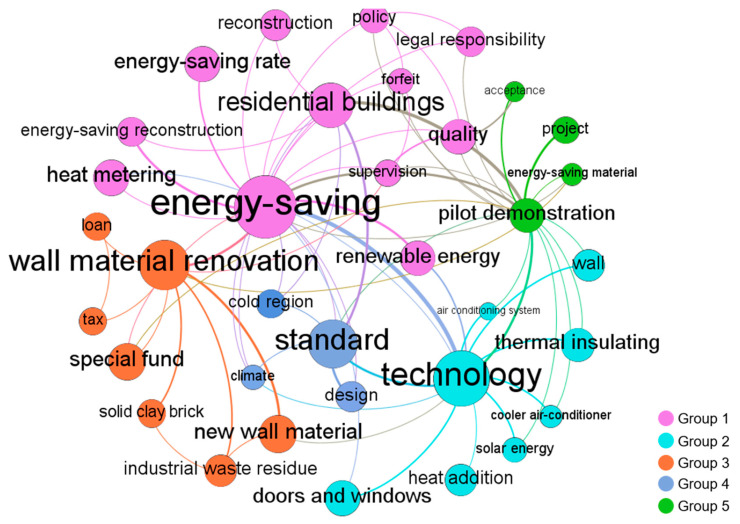
Co-word network of China’s GBPs in stage 1.

**Figure 3:**
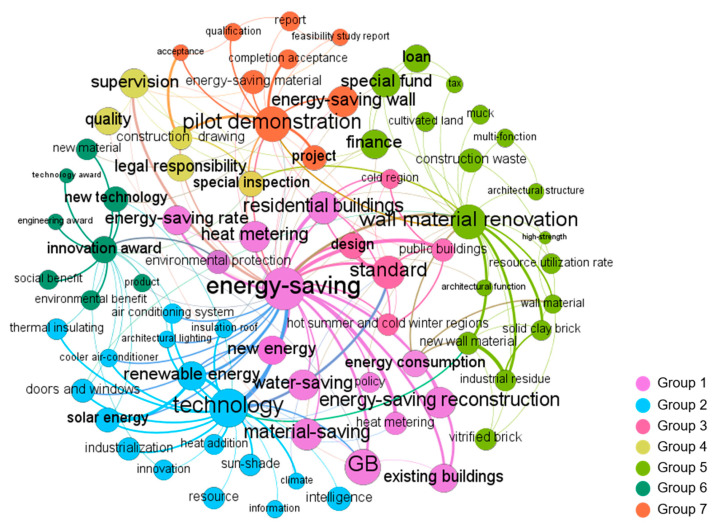
Co-word network of China’s GBPs in stage 2.

**Figure 4:**
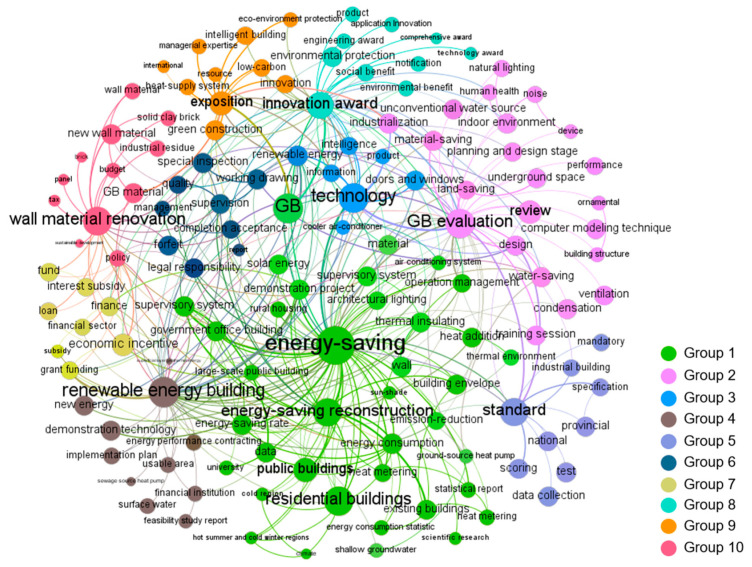
Co-word network of China’s GBPs in stage 3.

**Figure 5:**
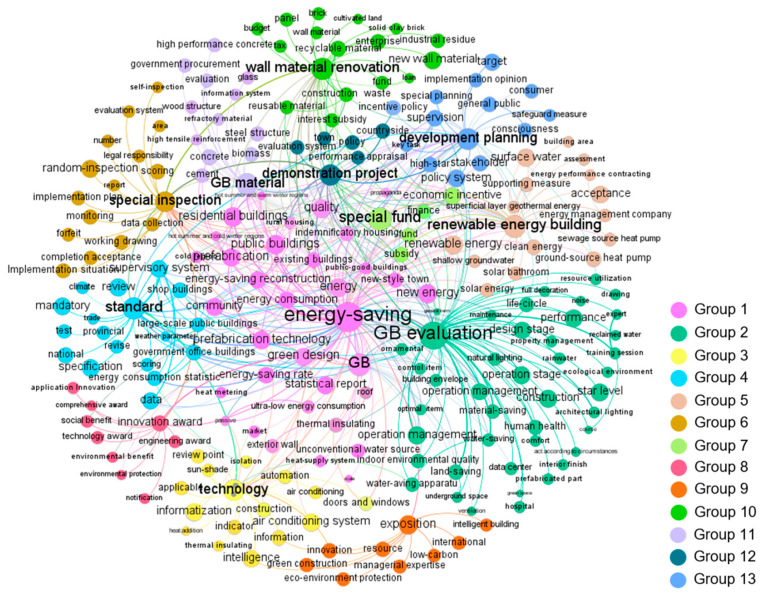
Co-word network of China’s GBPs in stage 4.

**Figure 6:**
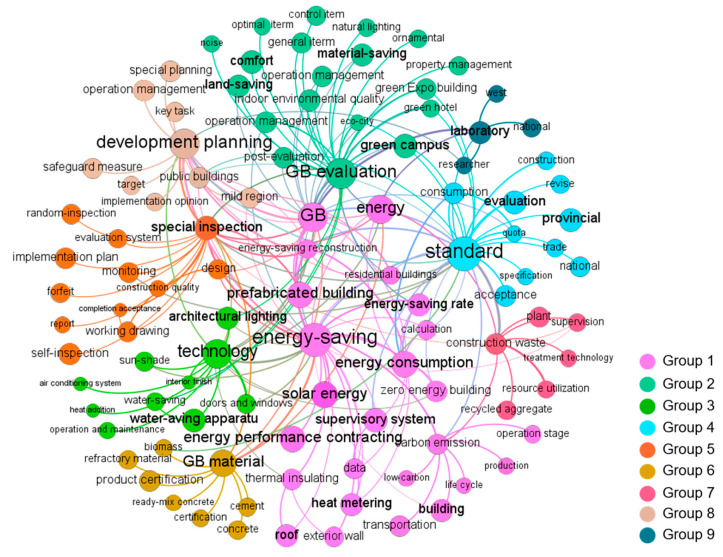
Co-word network of China’s GBPs in stage 5.

The co-word network graphs of each stage visualized by Gephi are shown in Figures 2–6. In these figures, the node represents the keyword and its size implies the word frequency. The lines and their thicknesses represent the co-occurrence relationship and co-occurrence intensity of the keywords, respectively. All keywords in the same cluster are displayed as nodes with the same color and are used to explain policy topics [51]. 

#### 3.1.1. Topics Discovered in Stage 1 (1986–2000)

The co-occurrence relationships of 35 keywords extracted from 13 GBPs of stage 1 are visualized in Figure 2. These keywords were clustered into five groups, representing the different GBP topics. It can be seen that “energy-saving,” “technology,” “wall material renovation,” “standard,” and “pilot demonstration” were the most important GBP topics in stage 1.

“Energy-saving,” the keyword in Group 1, had the highest frequency of occurrence in the co-word network. In 1994, MOHURD pointed out that “Building energy saving is the most direct and cheap fundamental measure to ease the energy shortage contradiction and reduce environmental pollution in China” and proposed that the energy-saving rate of new buildings should reach 50% by 2000 [56]. Thus, energy-saving was the main policy topic and GB performance indicator in stage 1. In this stage, the main object of GBPs was residential buildings, which was one of the high-frequency words in cluster 1. Technology was an important guarantee to achieve building energy-saving. In Group 2, “doors and windows,” “wall,” and “thermal insulating” were high-frequency keywords, indicating that energy-saving doors and windows and thermal insulation walls were the main energy-saving technology in stage 1. In Group 3, “wall material renovation” was identified as a high-frequency keyword. Due to new wall materials having potential advantages in the protection of cultivated land and the utilization of industrial waste residue, as early as 1992, the Chinese government issued the wall material innovation policy and implemented relevant financial incentive regulations such as special funds and tax reduction to promote the innovation of wall materials. In Group 4, “standard” was identified as a critical GBP topic. The Chinese government promulgated the first building energy-saving design standard in 1986 and revised it in 1995. This standard was only applicable to residential buildings in severe cold and cold regions of China—the keywords in Group 4 clearly reflect such a phenomenon. In Group 5, “pilot demonstration” had a lower frequency than other topic keywords, indicating that the pilot demonstration of green building has gradually attracted the attention of the government.

#### 3.1.2. Topics Discovered in Stage 2 (2001–2005)

A total of 76 keywords were extracted to identify the policy topics in this stage. As shown in Figure 3, these keywords were clustered into seven groups. Generally, the five policy topics of the previous stage continued to play an important role. In addition, “supervision” and “innovation award” were added in this stage. 

“Energy-saving” was still the most frequent keyword in this stage, which confirmed that it remained a key policy target. Different from the previous stage, “energy-saving” had strong linkages with new keywords “public buildings” and “existing buildings” in Group 1. In 2002, MOHURD [57] announced that during the 10th Five-Year Plan period, the foci of building energy-saving work were to promote the energy-saving transformation of existing buildings and promote the energy-saving of public buildings. Consistent with this announcement, the main objects of GBPs during stage 2 expanded from residential buildings to public buildings and existing buildings. “Technology” was identified as a high-frequency keyword in Group 2. In 2005, the first “Technical Guidelines for Green Buildings” was issued. In these guidelines, MOHURD clarified the meaning of GBs, the key points technology, and the performance indicator system of green building for the first time. Among them, GB technology and GB performance indicators mainly included five aspects: water-saving, energy-saving, material-saving, land-saving, and indoor environmental quality. In Group 3, “standard” was identified as a high-frequency keyword. Since there were few standards related to GB in stage 1, MOHURD issued some energy-saving design standards suitable for different climatic regions and different building types in this stage, such as the Energy-Saving Design Standards for Public Buildings. In 2002, MOHURD [57] stated that during the 10th Five-Year Plan period, the Regulations on the Management of Energy-Saving of Civil Buildings must be fully implemented, which was the first document in China to incorporate quality supervision and management into the policy [58]. Against this backdrop, “supervision” in Group 4 was identified as a critical policy topic, and this keyword was closely related to “construction drawing,” indicating that the government paid more attention to the supervision of the building design stage. In Group 5, “wall material renovation” was still a high-frequency keyword. Compared with the previous stage, “wall material renovation was closely related to the new keyword “architectural function.” By reviewing the policy documents in this stage, it was found that the Chinese government proposed to develop new wall materials with different functions to meet the needs of different building structures and functions. “Innovation award” was identified as a high-frequency keyword in cluster 6 because MOHURD issued the National Green Building Innovation Award in this stage to promote the development of GB and its technology. In Group 7, the high-frequency keyword “pilot demonstration” was identified as a critical policy topic. In light of the fact that pilot demonstration projects play an important role in accumulating technical experience and influencing neighboring construction projects to adopt sustainable measures, the Chinese government proposed to carry out numerous pilot demonstration projects for GB in this stage so as to promote the popularization of green building concepts and the development of green building technology.

#### 3.1.3. Topics Discovered in Stage 3 (2006–2010)

Regarding GBPs in stage 3, 138 keywords were extracted to identify the policy topics and were clustered into 10 groups. As shown in Figure 4, “energy-saving,” “GB evaluation,” “technology,” “renewable energy building,” “standard,” “supervision,” “special fund,” “innovation award,” “exposition,” and “wall material renovation” were the most frequent keywords in each group. 

In Group 1, “energy-saving” and “energy-saving reconstruction” increased compared with their frequency in the previous stage, which illustrated that the energy-saving reconstruction of existing buildings became the key policy target in this stage. For example, in the 11th Five-Year Plan Building Energy Conservation Task, the Chinese government clearly proposed that 150 million square meters of reconstruction area should be completed in 2010 [59]. In addition, an interesting finding is that “energy-saving” had strong linkages with the keywords “supervisory system” and “energy consumption,” which showed that the Chinese government began to focus on the application of modern technology to better manage the using of energy in high-energy buildings [60,61]. “GB evaluation” was a new high-frequency keyword. Influenced by countries leading the way in GB development, MOHURD issued China’s first green building evaluation standard in 2006. “Renewable energy building” was also a new high-frequency keyword in this stage. With the improvement of people’s life quality, the contradiction between energy supply and demand in buildings was becoming more and more serious. Promoting the development of renewable energy buildings in the building sector became the most economical and reasonable choice [60,61]. In 2006, MOHURD and Ministry of Finance (MOF) [62] issued the “Implementation Opinions on Promoting the Application of Renewable Energy in the building sector,” which clearly proposed that the policy and regulation system of renewable energy buildings should be basically formed in this stage. Against this backdrop, a large number of policies related to renewable energy building were issued. In Group 7, “special funds” was identified as a high-frequency keyword because the government began to attach importance to the financial incentive policies during this period and provide financial support for the building projects that met green building requirements. In 2010, the exposition initiated by MOHURD was held in China. According to the keywords in Group 9, it can be seen that the “exposition” mainly discussed the latest achievements, development trends, new technologies, and new products of GBs at home and abroad. This provided a learning platform for China to produce independent innovation technology.

#### 3.1.4. Topics Discovered in Stage 4 (2011–2015)

In stage 4, 218 keywords were used to identify policy topics, and these keywords were divided into 13 groups, as shown in Figure 5. “GB material,” “demonstration project,” and “development planning” were new high-frequency keywords, reflecting three emerging topics of the GBPs in stage 4. The policy topics reflected by the remaining high-frequency keywords were similar to those in stage 3. 

“Energy-saving” was still the highest frequency of occurrence in the co-word network. It is worth noting that “energy-saving” had a strong linkage with some new keywords in Group 1, including “industrialization,” “prefabrication technology,” and “prefabrication.” Due to the advantages of saving material and protecting the environment, prefabrication technology has been identified as an effective technique to improve the environmental performance of buildings. In the “Implementation Opinions on Accelerating the Development of Green Buildings in China,” MOHURD and MOF [63] mentioned promoting housing industrialization and promoting the use of prefabrication technology in buildings. In the “Green Building and Green Ecological Urban Development Planning” (hereinafter referred to as the “Planning”), MOHURD [64] proposed six suggestions to accelerate the development of the GB industry during the 12th Five-Year Plan period, including the use of prefabrication technology. In conclusion, similar to the study of Wang et al. [31], a strong linkage between “green building” and “prefabrication technology” appeared in stage 3. In addition, in the “Planning,” MOHURD [64] proposed to formulate green building evaluation standards suitable for different climate regions and building types during the 12th Five-Year Plan period. Therefore, in Group 4, the keyword “standard” had a strong linkage with “hospitals,” “data center buildings,” and “existing buildings.” In Group 5, the keyword “renewable energy building” was connected with some new keywords such as “energy management company” and “energy contract management,” which reflected that the Chinese government was trying to promote energy-saving and emission reduction in the building sector through a market mechanism. In Group 11, “GB material” was identified as a high-frequency keyword that had a strong linkage with “informatization.” In recent years, the Chinese government began to pay attention to the establishment of a GB material traceability information system by using two-dimensional code, cloud computing, and other technologies so as to improve the level of GB material logistics informatization and supply chain coordination.

#### 3.1.5. Topics Discovered in Stage 5 (2016–2019)

In the last stage, 104 keywords were clustered into nine topics (see Figure 6): energy-saving, GB evaluation, technology, standard, supervision, GB material, construction waste, development planning, and laboratory. 

“Energy-saving” remained the highest frequency unchanged. But different from the previous stage, “energy-saving” had a strong linkage with the new keywords “zero energy consumption building” and “mild region.” In 2017, MOHURD [65] first mentioned zero energy consumption buildings [66], and released the Near Zero Energy Consumption Building Technical Standard, which indicated that a higher target for China’s building energy-saving was established. In 2019, MOHURD issued the Design Standard for Energy Efficiency of Residential Buildings in the Mild Region. So far, China’s design standards for building energy-saving covered all climatic regions of the country. In Group 2, the high-frequency keyword “GB evaluation” had a strong linkage with “green campus,” “green hotel,” and “post evaluation,” which showed that the objects of GBPs were further expanded and that the Chinese government paid more attention to the quality supervision of GB evaluation projects in the operation stage. The keyword “technology” in Group 3 had a strong linkage with “operation and maintenance.” In 2016, MOHURD issued the Technical Specification for Green Building Operation and Maintenance, which stipulated the key operation technologies and established the evaluation index system of GB operation and maintenance. The release of this policy once again confirmed that the Chinese government attached great importance to the operation stage of green building. The keyword “prefabricated building” and some high-frequency keywords increased compared with their frequency in the previous stage, such as “development planning” and “supervision.” In light of the advantages of prefabricated buildings, the Chinese government gradually realized the importance of prefabricated technology in promoting GBD. Since 2016, the Chinese government has repeatedly proposed to promote the integrated development of GBs and prefabricated buildings, and has formulated corresponding regulatory policies. “Construction waste” was a new high-frequency keyword, which had a strong linkage with “resource utilization” and “standard.” With the rapid development of urban construction in China, more and more construction waste was produced in the process of urban demolition, housing construction, and decoration [67]. In the “Outline of the 13th Five-Year Plan,” the State Council (SC) proposed to promote the resource utilization of construction waste. Subsequently, the Chinese government issued numerous policies related to construction waste to promote the healthy development of the whole construction waste treatment industry, such as the Industrial Standard for the Resource Utilization of Construction Waste. Against this backdrop, construction waste became a critical policy topic and a new performance indicator for GB in this stage. In Group 10, “laboratory” was a new emerging keyword. In 2018, the State Key Laboratory of GB in Western China was established in Shaanxi province, creating conditions for training high-level GB researchers.

### 3.2. GB Policymaking Agencies

A review of the GBPs issued from 1986 to 2019 showed that a total of 14 agencies participated, as shown in Table 1. Since the names of some government agencies have changed in the past 34 years, to avoid confusion, similar to the study by Wang et al. [31], this paper adopted the latest names of these agencies. 

The number of different government agencies participating in the GBP release is also presented in Table 1. It can be found that the Ministry of Housing and Urban-Rural Development (MOHURD) was the core policymaking agency, and its participation in issuing policies accounted for 90.45% of the total policies. In China, one of the responsibilities of MOHURD is to promote building energy-saving and emission reduction. As GB is not only a kind of building type but also a key development object of building energy-saving and emission reduction, MOHURD plays an important role in the formulation of GBP. The Ministry of Finance (MOF), the Ministry of Industry and Information Technology (MIIT), and the Ministry of Science and Technology (MOST) each published at least nine policies. MOF is responsible for formulating policies on special funds and financial subsidies for GB. MIIT formulates industry planning, policies, and standards, and guides information construction. MOST is mainly responsible for the formulation of planning, policies, and measures related to innovation and advanced technology.

**Figure 7:**
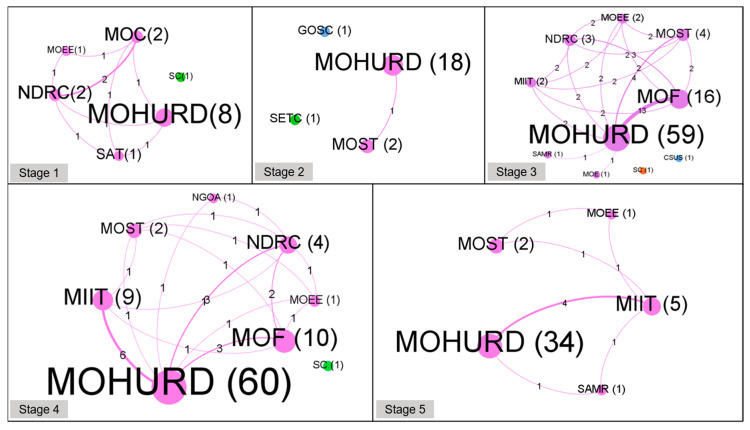
Co-author networks of GB policymaking agencies in five stages.

The cooperation networks of policymaking agencies in different stages are shown in Figure 7. In this figure, the nodes represent the policymaking agencies, and the size of the node represents the number of policies that the policymaking agency participates in releasing. The edges represent the cooperation relationships among these policymaking agencies, which are quantified by the number of collaborative GBP documents between each pair of nodes. The dynamics of these co-author network analyses reflect the historical variations of policymaking agencies as well as their evolving roles and powers in policymaking [43]. 

In stage 1, a total of six government agencies contributed to policy release, and four of them cooperated closely because they jointly released the “Design Standard for Energy-Saving of Civil Buildings (Heating Residential Buildings).”

In stage 2, the number of government agencies decreased to four, and their cooperative relationships were loose. In the above two stages, due to China’s GB being still in its development infancy, the number of policy agencies involved in policymaking was relatively small and their partnerships were simple.

In stage 3, the number of government agencies increased significantly, and their cooperative relationships became complex and close, which showed that the Chinese government began to pay more attention to the GBD. In addition, it can be found that MOHURD and MOF had the closest cooperation, indicating that the Chinese government tried to stimulate the enthusiasm of the market to develop GBs through the promulgation of financial incentive policies in this stage.

In stage 4, the number of agencies decreased, whereas their collaborative relationships became closer. An interesting finding is that policies issued by MOHURD alone in this stage account for a higher proportion than those issued by MOHURD alone in the previous stage, which showed that MOHURD’s centralized responsibility in GB policymaking began to return. In addition, MOHURD and MIIT had the closest cooperative relationships, as numerous industry planning and standards were issued in this stage.

In stage 5, the number of government agencies was further reduced, and their cooperative relationships became simpler. Among these connections, the alliance between MOHURD and MOF was eliminated and the interactions between MOHURD and MIIT became closer, indicating that the Chinese government mainly promulgated some standards and planning in this stage. In addition, 94.44% of the policies were released by MOHURD, implying the return of centralized responsibility in GB policymaking.

## 4. Discussion

Based on the above results and primary analyses, the historical dynamics of GBPs are further considered from five aspects: policy targets, policy objects, policy instruments, GB performance indicators, and the collaboration structure of policymaking agencies, as illustrated in Table 2. The policy targets were mainly extracted from the significant policy documents in each stage, such as the “Outline of the 10th Five-Year Plan for Building Energy Saving.” The policy instruments, including direction-based policies (DP), regulation-based policies (RP), evaluation-based policies (EP), financial support policies (FP), supervision policies (SP), organization and professional training (OP), and knowledge and information (KI), were employed in this study. Referring to previous studies [37,68], 10 GB performance indicators were adopted such as quality, energy, CO_2_ emissions, and water (see Table 2 for details).

Based on the analysis results, it can be seen that China’s GB originated from implementing energy-saving technologies. At the end of the 20th century, building energy-saving was considered to be the most direct and fundamental measure for alleviating the energy shortage contradiction and reducing environmental pollution [56]. Therefore, the main target of China’s early GBP target was energy-saving. For example, in the “Design Standard for Energy-Saving of Civil Buildings (Heating Residential Buildings),” the Chinese government proposed that the energy-saving rate of buildings should reach 50%. With the rapid development of the economy and the acceleration of urbanization, the building energy demand was also increasing. Thus, the Chinese government first increased the building energy-saving target from 50% to 65%, and then raised the building energy-saving target to 75% [69]. In addition, in stage 4, MOHURD [25] had formulated four other detailed policy targets, including the implementation of the demonstration construction of 100 green ecological cities and the promotion of energy-saving reconstruction of existing buildings. In stage 5, the targets of GBPs included the improvement of the GB standard system and the further expansion of GB application scale [65].

The main object of China’s GBP was residential buildings before 2000 and in the following year, MOHURD [57] proposed to strengthen the energy-saving work of residential buildings and public buildings as well as to promote the energy reconstruction of existing buildings. With the substantial increase in the numbers of GBPs in stage 3, the policy objects were further extended, including government office buildings, large-scale public buildings with high energy consumption, industrial buildings, universities, and rural housing. In stage 4, to explore the sustainable development model in the process of urbanization, the Chinese government issued the “National New Urbanization Plan (2014–2020),” which proposed to develop green towns and green urban areas. In stage 5, the policy objects had been continuously refined, including green hotel building, green exhibit building, etc.

From 1986 to 2019, China’s GBPs always included an RP instrument, DP instrument, and FP instrument. With its expertise in promoting technological progress and standardizing market order, the RP instrument was the most widely employed GBP instrument and received increasing attention from the Chinese government. Second to the RP instrument, the DP instrument was also highly valued by the Chinese government because it played a critical role in summarizing the development experience of GB and planning the development direction of GB. The FP instrument was also usually employed by the Chinese government due to its expertise in mobilizing the market to actively implement GBs. Based on the existing policy instruments in stage 1, the SP instrument and KI instrument were added in stage 2, indicating that the Chinese government began to attach importance to the use of a mandatory supervision policy to regulate the implementation of GBs. In stage 3, the number of policies under the KI instrument increased, which showed that the Chinese government attached great importance to enhancing public awareness of GB. The policy instruments in stage 4 remained unchanged, whereas the FP instrument was absent in stage 5, which shows that China’s green building is well on the way to becoming mandatory for all construction projects, rather than a socially conscious, idealistic option [20].

The early GBPs were devoted to building energy-saving, which was regarded as a means for addressing the energy shortage. Furthermore, building energy-saving mainly focused on the thermal insulation of the building envelope brought on by the use of new wall materials. Therefore, the early GB performance indicators mainly consisted of energy, quality, and employment of new wall materials. In stage 2, energy was still the most important performance indicator of GBs because the Chinese government proposed to ease the pressure on building energy demand by increasing the use of new and renewable energy. In addition, the performance indicators of this stage also included water, lad, material, and indoor environmental quality, which were first mentioned by MOHURD in “Technical Guidelines for Green Building.” In stage 3, construction waste was mentioned by more and more policies, such as China’s first GB evaluation standard and the “Notice on Supervision of Building Energy Saving,” which has been regarded as an emerging indicator to measure the performance of GBs. In stage 4, an emerging indicator, the employment of innovation and advanced technology, was utilized to measure the performance of GBs. Specifically, these technologies included two-dimensional code, a building information model (BIM), prefabrication technology, and so on. In stage 5, China’s GB evaluation standard was revised again in 2019, and the GB performance indicators changed from focusing on saving resources and environmental protection to being people-oriented. Therefore, health and well-being were recognized as additional emerging GB performance indicators.

From 1986 to 2019, there were several changes in the number of GB policymaking agencies and their cooperative relationships in China. From stage 1 to stage 2, the number of GB policymaking agencies in China decreased slightly, and their cooperative relationship tended to be simple. In stage 3, the number of policymaking agencies increased, and then they decreased again stage 4 and in stage 5. Meanwhile, the collaboration network of policymaking agencies went from dispersive to centralized, and MOHURD was the core institution with the largest number of policies issued in each stage.

## 5. Conclusions

A systematic and insightful investigation of the dynamic evolution of GBPs is of great significance for understanding of GBP background. This study investigated the development dynamics of GBPs in China from 1986 to 2019 by mapping and visualizing the co-word network and co-author network. The derived results revealed that China’s GBPs have evolved through five stages: theoretical exploration, pilot demonstration, rapid growth, scope expansion, and deepening development. Throughout GBP development, energy-saving has always been the main policy topic and the target of most concern in the past decades. Meanwhile, the topics related to material saving and construction waste management emerged in recent years. Though energy was always the main GB performance indicator, the GBPs began to pay attention to the indicators concerning health and well-being. In addition, it was revealed that MOHURD played an important role in the development of GBPs in China.

The investigation of the dynamic evolution of China’s GBPs can facilitate the stakeholders from both China and overseas in promoting GB practice. For the Chinese stakeholders, a clear understanding of the policy dynamics can help them to address the most innovative GB technologies and the most recent incentive measures. For the overseas stakeholders, the findings can provide valuable insights for countries in which the basic economic development and building sector conditions are similar to those in China. However, it should be mentioned that this study had specific limitations. For example, only national-level GBPs of China are considered in this study, whereas the GBPs of different cities in China are ignored. In fact, the historical dynamic of China’s local policies varies with the level of the local economy and are recommended for future studies.

## Figures and Tables

**Table 1:** Green building (GB) policymaking agencies in China from 1986 to 2019.

Government Department	Abbreviations	Description	No. of GBPs
Ministry of Housing and Urban-Rural Development	MOHURD	Government department	180
Ministry of Finance	MOF	Government department	25
Ministry of Industry and Information Technology	MIIT	Government department	16
Ministry of Science and Technology	MOST	Government department	9
National Development and Reform Commission	NDRC	Government department	8
Ministry of Ecology and Environment	MOEE	Government department	4
State Council	SC	The highest organ of state administration	4
State Administration of Market Regulation	SAMR	Government department	3
Ministry of Commerce	MOC	Government department	2
State Economic and Trade Commission	SETC	Government department	1
Chinese Society of Urban Science	CSUS	Social organization	1
State Administration of Taxation	SAT	Government department	1
National Government Offices Administration	NGOA	Government department	1
Ministry of Education	MOE	Government department	1

**Table 2:** Comparison of GBPs in different stages.

	Stage 1 (1986–2000)	Stage 2 (2001–2005)	Stage 3 (2006–2010)	Stage 4 (2011–2015)	Stage 5 (2016–2019)
**Policy targets**	Building energy-saving rate is 50% Wall material renovation	Building energy-saving rate is 65% Wall material renovation	More than 95% of the newly built buildings in cities and towns implement the mandatory energy-saving standards Promotion of the development of renewable energy buildings The application area of solar energy and shallow ground energy accounts for more than 25% of the new building area	Implementation of demonstration construction of 100 green ecological urban areas GB standards be implemented for government invested buildings, large public buildings, and commercial real estate projects Carrying out of energy-saving reconstruction of existing buildings	Improvement of building energy-saving standards Carrying out of energy-saving reconstruction of existing buildings Expansion of the application scale of renewable energy buildings Promotion of energy-saving of rural buildings
**Policy objects**	Residential buildings	Residential buildings Public buildings	Public buildings Residential buildings Government office buildings Large-scale public buildings Colleges and universities Industrial buildings	Residential buildings Public buildings Government office buildings Large-scale public buildings Public welfare buildings (schools, hospitals, etc.) Industrial buildings Indemnificatory housing New town Urban area Rural housing	Residential buildings Public buildings Government office buildings Large-scale public buildings Public welfare buildings (schools, hospitals, etc.) Industrial buildings Indemnificatory housings Rural housing New urban buildings Community (urban area) Office and store buildings Hotel buildings Prefabricated buildings
**Policy instruments**	DP/RP	DP/RP/FP/SP/KI	DP/RP/EP/FP/SP/OP/KI	DP/RP/EP/FP/SP/OP/KI	DP/RP/EP/FP/SP/OP/KI
**GB performance indicators**	Energy Employment of new wall materials Quality	Energy Employment of new wall materials Quality	Energy Quality Water GB materials Lad Employment of new wall materials CO_2_ emissions Construction waste	Energy Quality GB materials Water Lad Employment of innovation and advanced technology CO_2_ emissions Construction waste	Energy Quality GB materials Employment of innovation and advanced technology Health and well-being Water Lad Construction waste CO_2_ emissions
**Collaboration structure**	Some agencies with close collaboration	Few agencies with simple collaboration	More agencies with complex and close collaboration	More agencies with decreased and decentralized collaboration	Few agencies with centralized collaboration

DP: Direction-based policies, RP: Regulation-based policies, FP: Financial support policies, SP: Supervision policies, KI: Knowledge & information, EP: Evaluation-based policies, OP: Organization & professional training.

## References

[1] Li Y.A., Yang L., He B.J., Zhao D.D. (2014). Green building in China: Needs great promotion. Sustain. Cities Soc..

[2] Zuo J., Zhao Z. (2014). Green building research-current status and future agenda: A review. Renew. Sustain. Energy Rev..

[3] Wu Z., Yu A.T.W., Poon C.S. (2019). An off-site snapshot methodology for estimating building construction waste composition—A case study of Hong Kong. Environ. Impact Assess. Rev..

[4] Tang E.Z., Peng C., Xu Y.L. (2018). Changes of energy consumption with economic development when an economy becomes more productive. J. Clean. Prod..

[5] Sun H., Edziah B.K., Sun C., Kporsu A.K. (2019). Institutional quality, green innovation and energy efficiency. Energy Policy.

[6] Scherer A.G., Palazzo G., Seidl D. (2013). Managing legitimacy in complex and heterogeneous environments: Sustainable development in a globalized world. J. Manag. Stud..

[7] Varma C.R.S., Palaniappan S. (2019). Comparision of green building rating schemes used in North America, Europe and Asia. Habitat Int..

[8] Opoku A. (2019). Biodiversity and the built environment: Implications for the sustainable development goals (SDGs). Resour. Conserv. Recycl..

[9] Singha R., Samanta S., Chatterjee S., Pariari A., Majumdar D., Satpati B., Wang L., Singha A., Mandal P. (2018). Probing lattice dynamics and electron-phonon coupling in the topological nodal-line semimetal ZrSiS. Phys. Rev. B.

[10] Wu Z., Yu A.T.W., Wang H., Wei Y., Huo X. (2019). Driving factors for construction waste minimization: Empirical studies in Hong Kong and Shenzhen. J. Green Build..

[11] Santamouris M. (2016). Innovating to zero the building sector in Europe: Minimising the energy consumption, eradication of the energy poverty and mitigating the local climate change. Sol. Energy.

[12] Wen B., Musa S.N., Onn C.C., Ramesh S., Liang L., Wang W., Ma K. (2020). The role and contribution of green buildings on sustainable development goals. Build. Environ..

[13] Vyas G.S., Jha K.N. (2018). What does it cost to convert a non-rated building into a green building?. Sustain. Cities Soc..

[14] Wu Z., Jiang M., Cai Y., Wang H., Li S. (2019). What Hinders the Development of Green Building? An Investigation of China. Int. J. Environ. Res. Public Health.

[15] Wei Y., Zhu X., Li Y., Yao T., Tao Y. (2019). Influential factors of national and regional CO_2_ emission in China based on combined model of DPSIR and PLS-SEM. J. Clean. Prod..

[16] Bao Z., Lee W.M., Lu W. (2020). Implementing on-site construction waste recycling in Hong Kong: Barriers and facilitators. Sci. Total Environ..

[17] Darko A., Zhang C., Chan A.P.C. (2017). Drivers for green building: A review of empirical studies. Habitat Int..

[18] Zhang J., Li H., Olanipekun A.O., Bai L. (2019). A successful delivery process of green buildings: The project owners’ view, motivation and commitment. Renew. Energy.

[19] Balaban O., Puppim de Oliveira J.A. (2017). Sustainable buildings for healthier cities: Assessing the co-benefits of green buildings in Japan. J. Clean. Prod..

[20] Olubunmi O.A., Xia P.B., Skitmore M. (2016). Green building incentives: A review. Renew. Sustain. Energy Rev..

[21] Shan M., Hwang B.-g. (2018). Green building rating systems: Global reviews of practices and research efforts. Sustain. Cities Soc..

[22] Sharma M. (2018). Development of a ‘green building sustainability model’ for green buildings in India. J. Clean. Prod..

[23] Liang X., Yu T., Guo L. (2017). Understanding Stakeholders’ Influence on Project Success with a New SNA Method: A Case Study of the Green Retrofit in China. Sustainability.

[24] Zhang Y., Kang J., Jin H. (2018). A review of green building development in China from the perspective of energy saving. Energies.

[25] MOHURD 12th Five-Year Plan for Green Building Development Plan. http://www.gov.cn/gzdt/2013-04/18/content_2380994.htm.

[26] Wu Z., Liu L., Li S., Wang H. (2020). Investigating the Crucial Aspects of Developing a Healthy Dormitory based on Maslow’s Hierarchy of Needs—A Case Study of Shenzhen. Int. J. Environ. Res. Public Health.

[27] Chang R.d., Soebarto V., Zhao Z.y., Zillante G. (2016). Facilitating the transition to sustainable construction: China’s policies. J. Clean. Prod..

[28] Shi Q., Lai X., Xie X., Zuo J. (2014). Assessment of green building policies-A fuzzy impact matrix approach. Renew. Sustain. Energy Rev..

[29] Liu G., Tan Y., Li X. (2020). China’s policies of building green retrofit: A state-of-the-art overview. Build. Environ..

[30] Ye L., Cheng Z., Wang Q., Lin H., Lin C., Liu B. (2015). Developments of green building standards in China. Renew. Energy.

[31] Wang Y.N., Xue X.L., Yu T., Wang Y.W. (2020). Mapping the dynamics of China’s prefabricated building policies from 1956 to 2019: A bibliometric analysis. Build. Res. Inf..

[32] Dou Y., Xue X., Wang Y., Luo X., Shang S. (2019). New media data-driven measurement for the development level of prefabricated construction in China. J. Clean. Prod..

[33] Darko A., Chan A.P.C. (2016). Critical analysis of green building research trend in construction journals. Habitat Int..

[34] Darko A., Chan A.P.C., Huo X., Owusu-Manu D.-G. (2019). A scientometric analysis and visualization of global green building research. Build. Environ..

[35] Dat Tien D., Ghaffarianhoseini A., Naismith N., Zhang T., Ghaffarianhoseini A., Tookey J. (2017). A critical comparison of green building rating systems. Build. Environ..

[36] Liu X., Wang M., Fu H. (2020). Visualized analysis of knowledge development in green building based on bibliographic data mining. J. Supercomput..

[37] Zhang Y., Wang J., Hu F., Wang Y. (2017). Comparison of evaluation standards for green building in China, Britain, United States. Renew. Sustain. Energy Rev..

[38] Yuan X., Zuo J. (2011). Transition to low carbon energy policies in China-from the Five-Year Plan perspective. Energy Policy.

[39] Hu A.-G. (2016). The Five-Year Plan: A new tool for energy saving and emissions reduction in China. Adv. Clim. Chang. Res..

[40] Zupic I., Cater T. (2015). Bibliometric methods in management and organization. Organ. Res. Methods.

[41] Yang C., Huang C., Su J. (2020). A bibliometrics-based research framework for exploring policy evolution: A case study of China’s information technology policies. Technol. Forecast. Soc. Chang..

[42] Zhang Q., Lu Q., Zhong D., Ye X. (2018). The pattern of policy change on disaster management in China: A bibliometric analysis of policy documents, 1949–2016. Int. J. Disaster Risk Sci..

[43] Huang C., Su J., Xie X., Ye X., Li Z., Porter A., Li J. (2015). A bibliometric study of China’s science and technology policies: 1949–2010. Scientometrics.

[44] Khanra S., Dhir A., Mantymaki M. (2020). Big data analytics and enterprises: A bibliometric synthesis of the literature. Enterp. Inf. Syst..

[45] Feng J., Zhang Y.Q., Zhang H. (2017). Improving the co-word analysis method based on semantic distance. Scientometrics.

[46] Feng Y., Zhu Q., Lai K.-H. (2017). Corporate social responsibility for supply chain management: A literature review and bibliometric analysis. J. Clean. Prod..

[47] Du H., Li N., Brown M.A., Peng Y., Shuai Y. (2014). A bibliographic analysis of recent solar energy literatures: The expansion and evolution of a research field. Renew. Energy.

[48] Du H., Li B., Brown M.A., Mao G., Rameezdeen R., Chen H. (2015). Expanding and shifting trends in carbon market research: A quantitative bibliometric study. J. Clean. Prod..

[49] Li J., Wang M., Ho Y. (2011). Trends in research on global climate change: A science citation index expanded-based analysis. Glob. Planet. Chang..

[50] Nan Y., Feng T., Hu Y., Qi X. (2020). Understanding aging policies in China: A bibliometric analysis of policy documents, 1978–2019. Int. J. Environ. Res. Public Health.

[51] Yao H., Zhang C. (2018). A bibliometric study of China’s resource recycling industry policies: 1978–2016. Resour. Conserv. Recycl..

[52] Kauffman J., Kittas A., Bennett L., Tsoka S. (2014). DyCoNet: A Gephi plugin for community detection in dynamic complex networks. PLoS ONE.

[53] Wang X.L., Liu Y., Ju Y.B. (2018). Sustainable public procurement policies on promoting scientific and technological innovation in China: Comparisons with the US, the UK, Japan, Germany, France, and South Korea. Sustainability.

[54] Ding Z., Liu R., Li Z., Fan C. (2020). A thematic network-based methodology for the research trend identification in building energy management. Energies.

[55] Ding Z.K., Li Z.J., Fan C. (2018). Building energy savings: Analysis of research trends based on text mining. Autom. Constr..

[56] MOHURD Ninth Five-Year Plan and the 2010 Plan for Building Energy-Saving. http://www.cnki.com.cn/Article/CJFDTotal-SGJS608.000.htm.

[57] MOHURD Outline of the 10th Five-Year Plan for Building Energy Saving. http://www.mohurd.gov.cn/wjfb/200611/t20061101_158478.html.

[58] Ma M., Cai W., Wu Y. (2019). China act on the energy efficiency of civil buildings (2008): A decade review. Sci. Total Environ..

[59] MOHURD 11th Five-Year Plan for Building Energy Conservation Task. http://www.gov.cn/gzdt/2010-05/18/content_1608438.htm.

[60] Kong X., Lu S., Wu Y. (2012). A Review of Building Energy Efficiency in China during “Eleventh Five-Year Plan” Period. Energy Policy.

[61] Guo Q., Wu Y., Ding Y., Feng W., Zhu N. (2016). Measures to enforce mandatory civil building energy efficiency codes in China. J. Clean. Prod..

[62] MOHURD, MOF Implementation Opinions on Promoting the Application of Renewable Energy in the Building Sector. http://www.mohurd.gov.cn/wjfb/200611/t20061101_158510.html.

[63] MOHURD, MOF Implementation Opinions on Accelerating the Development of Green Buildings in China. http://www.mohurd.gov.cn/fgjs/xgbwgz/201205/t20120510_209831.html.

[64] MOHURD Green Building and Green Ecological Urban Development Planning. http://www.mohurd.gov.cn/wjfb/201304/t20130412_213405.html.

[65] MOHURD 13th Five-Year Plan for Building Energy Saving and Green Buildings Development. http://www.mohurd.gov.cn/wjfb/201703/t20170314_230978.html.

[66] Yang X., Zhang S., Xu W. (2019). Impact of zero energy buildings on medium-to-long term building energy consumption in China. Energy Policy.

[67] Wu Z., Yu A.T.W., Shen L. (2017). Investigating the determinants of contractor’s construction and demolition waste management behavior in Mainland China. Waste Manag..

[68] Geng Y., Ji W., Wang Z., Lin B., Zhu Y. (2019). A review of operating performance in green buildings: Energy use, indoor environmental quality and occupant satisfaction. Energy Build..

[69] Li J., Shui B. (2015). A comprehensive analysis of building energy efficiency policies in China: Status quo and development perspective. J. Clean. Prod..

